# Current Practices and Perceived Role of Community Pharmacists in Type 2 Diabetes Services in Pakistan

**DOI:** 10.7759/cureus.37311

**Published:** 2023-04-08

**Authors:** Rasikh Arif, Ali Zeb Khan, Muhammad Hammad, Usman Ghani, Ratnasree Vaddepalli, Vivek Sanker

**Affiliations:** 1 Clinical Research, Al-Shifa Trust Eye Hospital, Rawalpindi, PAK; 2 Pharmacy, Shifa Tameer-E-Millat University Shifa College of Medicine, Islamabad, PAK; 3 Clinical Research, Aga Khan University Hospital, Karachi, PAK; 4 General Medicine, Angeles University Foundation, Angeles, PHL; 5 General Surgery, Noorul Islam Institute of Medical Science and Research (NIMS), Trivandrum, IND

**Keywords:** pakistan, perceived role, diabetes, pharmacy services, community pharmacist

## Abstract

Background

Diabetes mellitus is a chronic illness which is becoming more prevalent in developing countries, and it is being managed mostly in hospitals or clinics in underdeveloped nations. Other strategies for treatment delivery in emerging nations must be considered as the number of diabetic patients grows. Community pharmacists are a valuable choice for diabetes care. However, only developed countries have data on community pharmacists' diabetes treatment practices.

Methodology

A non-probability consecutive sampling strategy was used to gather a self-administered questionnaire from 289 community pharmacists. Six points Likert scale was employed to score current practices and pharmacists' perceived role. A response rate of 55% was attained. Characteristics associated with present behaviors and perceived roles were analyzed using Chi-square and logistic regression.

Results

The majority of the respondents were males, 234 (81.0%). Out of 289, 229 (79.2%) were of 25-30 years of age and were pharmacists as well as qualified persons (QP) 189 (65.4%). A QP is one who has the legal authority to sell drugs to customers. The majority had <5 years of working experience as a community pharmacist, 268 (92.7%), and did not have diabetes training, 237 (82.0%). Most community pharmacies were stand-alone, 110 (38.1%), and had a single or a group of proprietors, 248 (85.8%). Open hours of most of the pharmacies were 16-20 hours per day, 202 (69.8%), and most had one pharmacist, 243 (84.1%), i.e., working as a pharmacist as well as a qualified person. Approximately 203 (70.2%) of the pharmacies had customers >2000 in a month and >100 customers purchased anti-diabetes medications per month. Only 44 (15.2%) community pharmacies had a designated room or space for patient counselling. The majority of pharmacists were also in favor of providing services other than dispensing such as counselling the patients about prescribed medicines, direction of use, use of devices for insulin administration, training on self-monitoring of glucose, and healthy lifestyle and diet practices. Pharmacy setting, ownership, patient counseling area, and the number of customers per month were key factors in the provision of diabetes services. The main obstacles identified were a lack of pharmacist availability and academic competency.

Conclusion

In Rawalpindi and Islamabad, most community pharmacies only provide a basic dispensing service for diabetes patients. Most of the community pharmacists agreed to extend their duties. The expansion of pharmacist professional responsibilities would help control the rising diabetes burden. The facilitators and hurdles identified would serve as a foundation for the introduction of diabetic care in community pharmacies.

## Introduction

Diabetes is among the most prevalent public health issues all over the world, and its prevalence is rising, especially in developing countries. Diabetes currently affects 415 million people globally, with that number estimated to rise to 642 million by 2040 [1]. When type 2 diabetes is combined with complications, it can have a significant impact on individuals, as well as societal implications. Because of growing urbanization, changes in diet, and a more sedentary lifestyle, diabetes has expanded to middle- and low-income nations, including Pakistan [2]. The number of patients with diabetes in Pakistan was 26.3% in 2016-2017, according to the National Diabetes Survey of Pakistan (NDSP) [3]. International Diabetes Federation (IDF) currently estimates that one in every 11 people has diabetes [4]. Moreover, 33 million adults in Pakistan have diabetes as of 2021, ranking it third in terms of the disease's burden [5]. Another study published in 2018 revealed that 16.98% of Pakistanis have type 2 diabetes [2]. Pakistan is dealing with a double burden of diseases, hunger, unemployment, illiteracy, as well as social and cultural aspects, all of which have resulted in a low priority being placed on disease prevention and its repercussions.

Many factors such as ineffective communication, counseling and limited time-sparing factors of the healthcare workers have contributed to the failure of effective management of chronic illnesses such as diabetes [6]. These gaps in management bring focus towards the participation of community pharmacists, which yielded positive outcomes in many settings [7].

The public has developed a misconception over the years that community pharmacies only sell medicines; however, the community pharmacist, as a custodian of the ailing community, can play a key role in changing this impression by providing health education about self-management, prevention of adverse effects, prevention of long-term complications, the direction of medication use, adherence, and therapeutic plan management. According to a Japanese study, community pharmacists’ consultations focusing on lifestyle changes helped patients with type II diabetes achieve better glycemic control. The patients' HbA1c levels were also observed to improve after 6 months of treatment, and the number of blood glucose-controlling medications was also reduced [8]. So, community pharmacists can be ideal additions to the multidisciplinary primary health care team as specialists in drug therapy, drug selection, patient education and counseling, leading to better care for the patients. Diabetes-oriented services at community pharmacies can help relieve the strain on other healthcare facilities by serving around 26% of the population. Diabetes care by community pharmacists is now confined to developed nations like the US, European countries, and the UK.

Therefore, the aim of the study is to evaluate existing practices and the community pharmacist's perceived role in type 2 diabetes care, as well as factors (pharmacist and community pharmacy) linked to the current practices.

## Materials and methods

In Rawalpindi and Islamabad, a cross-sectional (quantitative) study was undertaken to evaluate existing practices and the community pharmacist's perceived role in type 2 diabetes care, as well as factors (pharmacist and community pharmacy) linked to the current practices. These two cities were chosen as both cities' community pharmacy procedures are comparable. Rawalpindi has a population of 22.8 million people, whereas Islamabad has a population of roughly 11.6 million [9]. 289 community pharmacies were chosen at random from a list of registered pharmacies gathered from respected district health offices of Rawalpindi and Islamabad, Pakistan.

Sample size and setting

The study population was all community pharmacists working in community pharmacies of Rawalpindi and Islamabad. A non-probability consecutive sampling strategy was used to choose community pharmacies. The sample size was determined from a list of community pharmacies obtained from the respective health authorities. The total number of community pharmacies in Islamabad and Rawalpindi was 550 and 600, respectively (total 1150) [6]. A total of 289 responses were usable from the 525 questionnaires circulated as they were incomplete, producing a response rate of 55%, which was sufficient for statistical analysis. The sample size calculated using the OpenEpi version 3 sample size calculator at a 95% confidence interval was 289. 

Data collection

A 33-question pre-tested six-point Likert scale (see Appendices) on community pharmacy-based services and pharmacists' perceived involvement in type 2 diabetes services was used. An in-person survey was conducted with the help of a questionnaire. There were four sections to the research questionnaire: (1) Community pharmacist and Community pharmacy characteristics; (2) Current practices in the treatment of type 2 diabetes; (3) consent form, and (4) pharmacists' perceived roles for type 2 diabetes patients.** **The frequency of providing the services was scored using a six-point Likert scale; 1 (never) to 6 (often). The perceived role of the pharmacist in current diabetic practice was scored using a six-point Likert scale; 1 (definitely no) to 6 (definitely yes) Details of community pharmacists such as gender, age, position, experience as a community pharmacist, registration year, and duration of diabetes training were collected. And details about community pharmacies such as setting, opening hours per week, ownership, opening days per week, presence of counselling area/ room, number of pharmacists per pharmacy, customers per month, customers purchasing oral anti-diabetic medications and insulin per month were also collected. Informed and verbal consent was obtained from the community pharmacists only, no other healthcare practitioners or support workers were involved in the study. The responders were verbally informed of the confidentiality of the information, and the principal investigator signed a confidentiality undertaking, after getting formal approval from the Ethical Review Committee of Al-Shifa Trust Eye Hospital, School of Public Health in Rawalpindi (Reference no: ERC-13/AST-21).

Data analysis

Statistical Package for Social Science (SPSS) software version 26.0 (IBM Corp., Armonk, USA) and Microsoft Excel 365 (Microsoft Corporation, Redmond, USA) were used for statistical analysis and data entry. Data entry was done according to the codes assigned to all the items in the questionnaire, while numeric variables were segregated accordingly. The characteristics of the community pharmacists and their pharmacies were summarized using descriptive statistics. Frequencies were determined for responses from Likert scales of current practice and pharmacists' perceived roles in diabetes services. Current service responses were divided into two variables indicating ‘frequent service' (5-6 on the Likert scale) versus 'less frequent service' (1-4 on the Likert scale), as well as pharmacist roles, which were also divided into two variables indicating 'agreement' (5-6 on Likert scale) and 'disagreement' (1-4 on Likert scale). The frequencies of these replies were determined after coding. Chi-square test of association was used to determine the significance between independent and dependent variables. At 95% confidence intervals and p<0.05, a logistic regression model was developed to evaluate the relationship between features of community pharmacy and pharmacist with the current provision of services for diabetes and the perceived role of the community pharmacist. Tables are used to present the results.

## Results

Most respondents were male, 234 (81.0%). Out of 289, 229 (79.2%) were of 25-30 years of age and 189 (65.4%) were pharmacists as well as qualified persons (QP). The majority of community pharmacists, 268 (92.7%), had less than 5 years of experience. Five (1.7%) pharmacists had more than 10 hours of training, 13 (4.5%) had 6-10 hours of training, 34 (11.8%) had 1-5 hours of training, and 237 (82%) had no training in diabetes care. The majority of them had no training; however, in recent times pharmacists have been highly encouraged to undergo training as the role of pharmacists is expanding due to the increased number of patients being diagnosed with diabetes. Being trained definitely had an edge over not being trained in a few detailed aspects such as instructing the patients about the interaction of food consumed and blood sugar levels, monthly refilling of anti-diabetic drugs, meal planning etc. The majority of pharmacies were stand-alone, 110 (38.1%), and were owned by a single or group of entrepreneurs, 248 (85.8%). A total of 24 (8.3%) pharmacies were owned by trained pharmacist managers. 202 (69.9%) pharmacies were open for 15-20 hours a day, and 243 (84.1%) pharmacies had only one pharmacist in each pharmacy. To evaluate pharmacist availability in each community pharmacy, a ratio of the total working hours of a pharmacist in a week to the total operating hours of a pharmacy in a week was calculated. The ratios came out were 0.0-2.0, with the majority of pharmacies having less than 1.0 (80%). Thus, most of the pharmacies did not have a pharmacist on duty during the week while they were open. As the pharmacies hired only one pharmacist and they work for approximately 40 hours a week, most of them have their day off on weekdays. Approximately 203 (70.2%) pharmacies had >2000 monthly consumers, and 218 (75.4%) had >100 diabetic patients per month. Only 44 (15.2%) pharmacies had a separate room or area dedicated to counseling. Table 1 summarizes the characteristics of respondent pharmacists and their pharmacies in terms of frequencies and percentages.

**Table 1:** Characteristics of community pharmacists and community pharmacies (N = 289)

Characteristics of Community Pharmacist	Frequency	Percent
Gender		
Male	234	81.00%
Female	55	19.00%
Age		
<25 Years	37	12.80%
25-30 Years	229	79.20%
>30 Years	23	7.90%
Year of Registration		
<2015	22	7.60%
2015-2020	229	79.20%
>2020	38	13.14%
Position in community Pharmacy		
Pharmacist Owner and Manager of Community Pharmacy	24	8.30%
Pharmacist as a Manager of Community Pharmacy	24	8.30%
Pharmacist as well as Qualified Person (QP)	189	65.40%
Staff Pharmacist	52	18.00%
Number of years worked as a Community Pharmacist		
<5	268	92.70%
6-10	21	7.30%
Training for Diabetes Services		
None	237	82.00%
1-5 hours	34	11.80%
6-10 hours	13	4.50%
>10 hours	5	1.70%
Community Pharmacy Characteristics		
Type of Community Pharmacy		
Chain Store	110	38.10%
Stand Alone	108	37.40%
Community Pharmacy inside the shopping mall	8	2.80%
Community Pharmacy inside Clinics	63	21.80%
Proprietorship		
Pharmacist Owner and Manager of Community Pharmacy	24	8.30%
Single or group of entrepreneurs	248	85.80%
Non-Pharmacist Owner and Pharmacist Manager	17	5.90%
Number of working days in a week		
<7 Days	8	2.80%
7 Days	281	97.20%

Community pharmacists reported dispensing was a prominent service, but the additional diabetic services were minimal. Patient education on pharmaceuticals, notably directions for use 221 (76.5%), directions for use of insulin devices, and storage requirements 234 (81.0%) were the only services indicated by the majority of respondents.

All the community pharmacists were in favor of providing services other than dispensing and in expanding their staff. Counselling on prescribed medications like directions for use (99.7%) and use of devices for insulin administration (99.7%), training on self-monitoring of blood glucose (SMBG) (93.1%), education on healthy eating and exercise (91.3%), and strategy for prevention/treatment of long-term complications (91.3%) were the prime considerations for services other than dispensing (77.2%). Table 2 summarizes the responses regarding current practice and pharmacists' perceived roles in diabetes care.

**Table 2:** Current pharmacy-based practices and perceived role of community pharmacists (N = 289) BMI: body mass index, SMBG: Self-monitoring of blood glucose *Variable - Obtaining patient history such as age, diabetes duration and treatment, lifestyle, family history, and other cardiovascular risk factors, understanding about diabetes, past acute and chronic complications, psychological state, and record of other disorders **Variable - Participation in the treatment strategy: Personalized treatment goals, design treatment strategy that include antidiabetic drugs, exercise, diet plan, and the prevention and treatment of long-term complications. ***Variable - Referral, medication, and educational modifications were all part of the review.

Diabetes Services	Current Practices for type 2 diabetes in Community Pharmacies		Perceived role of Community Pharmacist for type 2 diabetes services	
	Being Frequently provided n (%)	Not Frequently provided n (%)	Agreement Perceived as ‘‘part of roles’’ n (%)	Disagreement Not Perceived as ‘‘part of roles’’ n (%)
Dispensing of Medications				
Prescription Filling	284 (98.3%)	5 (1.7%)	288 (99.7%)	1 (0.3%)
Labeling of medications for directions of use	284 (98.3%)	5 (1.7%)	288 (99.7%)	1 (0.3%)
Diabetes Services other than dispensing				
Initial evaluation				
Medical history of patient*	30 (10.4%)	259 (89.6%)	222 (76.8%)	67 (23.2%)
Basic medical evaluation (weight/height, blood pressure)	31 (10.7%)	258 (89.3%)	256 (88.6%)	33 (11.4%)
Check blood glucose level	31 (10.7%)	258 (89.3)	258 (89.3)	31 (10.7%)
Treatment plan**	8 (2.8%)	281 (97.2%)	233 (80.6)	56 (19.4%)
Patient education				
Disease process	16 (5.5%)	273 (94.5%)	228 (78.9%)	61 (21.1%)
Treatment targets	16 (5.5%)	273 (94.5%)	235 (81.3%)	54 (18.7%)
Antidiabetic medications:				
Directions for use	221 (76.5%)	68 (23%)	288 (99.7%)	1 (0.3%)
Use of insulin devices	234 (81.0%)	55 (19.0%)	288 (99.7%)	1 (0.3%)
Storage requirements	234 (81.0%)	55 (19.0%)	288 (99.7%)	1 (0.3%)
Special precautions to follow	108 (37.4%)	181 (62.6%)	251 (86.9%)	38 (13.1%)
Common/important adverse effects	55 (19.0%)	234 (81.0%)	251 (86.9%)	38 (13.1%)
Exercise	100 (34.6%)	189 (65.4%)	264 (91.3%)	25 (8.7%)
Diet	62 (21.5%)	227 (78.5%)	264 (91.3%)	25 (8.7%)
SMBG	61 (21.1%)	228 (78.9%)	269 (93.1%)	20 (6.9%)
Prevention/treatment of acute complications	38 (13.1%)	251 (86.9%)	265 (91.7%)	24 (8.3%)
Prevention/treatment of chronic complications	46 (15.9%)	243 (84.1%)	265 (91.7%)	50 (17.3%)
Needs for regular medical monitoring	47 (16.3%)	242 (83.7%)	239 (82.7%)	50 (17.3%)
Foot self-care	79 (27.3%)	210 (72.7%)	231 (79.9%)	58 (20.1%)
Smoking cessation	108 (37.4%)	181 (62.6%)	225 (77.9%)	64 (22.1%)
Monitoring				
Monitor compliance with				
Antidiabetic medications	20 (6.9%)	269 (93.1%)	141 (48.8%)	148 (51.2%)
Exercise plan	27 (9.3%)	262 (90.7%)	174 (60.2%)	115 (39.8%)
Diet plan	32 (11.1%)	257 (88.9%)	204 (70.6%)	85 (29.4%)
Strategy for prevention/treatment of long-term complications	32 (11.1%)	257 (88.9%)	223 (77.2%)	66 (22.8%)
Scheduled medical monitoring	32 (11.1%)	257 (88.9%)	223 (77.2%)	66 (22.8%)
Monitor treatment outcomes				
Check records on SMBG	31 (10.7%)	258 (89.3%)	212 (73.4%)	77 (26.6%)
Carry out blood glucose tests	47 (16.3%)	242 (83.7%)	215 (74.4%)	74 (25.6%)
Measure BMI	54 (18.7%)	235 (81.3%)	218 (75.4%)	71 (24.6%)
Measure blood pressure	63 (21.8%)	226 (78.2%)	207 (71.6%)	82 (28.4%)
Check results on laboratory tests	63 (21.8%)	226 (78.2%)	202 (71.6%)	87 (30.1%)
Monitor for adverse effects	23 (8.0%)	266 (92.0%)	233 (80.6%)	56 (18.4%)
Review ***	47 (16.3%)	242 (83.7%)	232 (80.3%)	57 (19.7%)

The association of pharmacists’ characteristics and the perceived role of community pharmacists in type 2 diabetes care are shown in Table 3. Chi-square results showed that the year of registration (p=0.000), pharmacy setting (p=0.004), ownership status of pharmacies (p=0.022), pharmacies' opening hours per day (p=0.004), patient counseling area/room (p=0.046), customer per month (p=0.000), customer purchasing oral anti-diabetic medication per month (p=0.000) and customer purchasing insulin per month (p=0.000) were significantly related to current type 2 diabetes services. On the other hand, age (p=0.033) and customer purchasing insulin per month (p=0.038) were the only factors that are significantly related to community pharmacists' perceived role in type 2 diabetes.

**Table 3:** Association of pharmacy and pharmacist characteristics and perceived role of community pharmacists for type 2 diabetes services

Pharmacist Characteristics	Current Type 2 Diabetes Services			Community Pharmacist perceived role for Type 2 diabetes services			
	Less Frequent Service n (%)	Frequent Service n (%)	P-0.05	Being Perceived as Role n (%)	Not being Perceived as Role n (%)		P-0.05
Gender							
Male	135 (57.7%)	99 (42.3%)	0.330	146 (62.4%)		88 (37.6%)	0.937
Female	23 (41.8%)	32 (58.2%)		34 (61.8%)		21 (38.2%)	
Age years							
<25 Years	54 (54.5%)	45 (45.5%)	0.975	70 (70.7%)		29 (29.3%)	0.033
>25 Years	104 (54.7%)	86 (45.3%)		110 (57.9%)		80 (42.1%)	
Year of Registration							
<2015	36 (81.8%)	8 (18.2%)	0.000	27 (81.8%)		17 (38.6%)	0.812
2015-2020	91 (44.0%)	116 (56%)		131 (63.4%)		76 (36.7%)	
>2020	31 (81.6%)	7 (18.4%)		180 (57.9%)		16 (42.1%)	
Position in community Pharmacy							
Pharmacist Owner and Manager of Community Pharmacy	7 (29.2%)	17 (70.8%)	0.065	15 (62.5%)		9 (37.5%)	0.459
Pharmacist as a Manager of Community Pharmacy	15 (62.5%)	9 (37.5%)		18 (75.0%)		6 (25.0%)	
Pharmacist as well as Qualified Person (QP)	106 (56.1%)	83 (43.9%)		118 (62.4%)		71 (37.6%)	
Staff Pharmacist	30 (57.7%)	22 (42.3%)		29 (55.8%)		23 (44.2%)	
Number of years worked as a Community Pharmacist							
<5	145 (54.1%)	123 (45.9%)	0.489	166 (61.9%)		102 (38.1%)	0.667
6-10	13 (61.9%)	8 (38.1%)		14 (66.7%)		109 (33.3%)	
Training for Diabetes Services							
None	126 (53.2%)	111 (46.8%)	0.448	141 (59.5%)		96 (40.5%)	0.075
<5 hours	22 (64.7%)	12 (35.3%)		27 (79.4%)		7 (20.6%)	
>5 hours	10 (55.6%)	8 (44.4%)		12 (66.7%)		6 (33.3%)	
Pharmacy Characteristics							
Setting							
Chain Store	71 (67.6%)	34 (32.4%)	0.004	60 (57.1%)		45 (42.9%)	0.160
Stand Alone	54 (47.8%)	59 (52.2%)		78 (69.0%)		35 (31.0%)	
Pharmacy within Shopping Mall Complex	33 (46.5%)	38 (53.5%)		42 (59.2%)		29 (40.8%)	
Ownership							
Pharmacist Manager as Owner	7 (29.2%)	17 (70.8%)	0.022	15 (62.5%)		9 (37.5%)	0.210
Single or Group Proprietor	143 (57.7%)	105 (42.3%)		151 (60.9%)		97 (39.1%)	
Partnership Proprietor-Pharmacist Manager	8 (47.1%)	9 (52.9%)		14 (82.4%)		3 (17.6%)	
Opening Days per week							
<7 Days	8 (53.3%)	7 (46.7%)	0.915	7 (46.7%)		8 (53.3%)	0.200
7 Days	150 (54.7%)	124 (45.3%)		173 (63.1%)		101 (36.9%)	
Opening Hours per Day							
<15 Hours	16 (66.7%)	8 (33.3%)	0.004	18 (75.0%)		6 (25.0%)	0.307
15-20 Hours	119 (58.9%)	83 (41.1%)		126 (62.4%)		76 (37.6%)	
>20 Hours	23 (36.5%)	40 (63.5%)		36 (57.1%)		27 (42.9%)	
Patient Counselling Area/room							
Yes	18 (40.9%)	26 (59.1%)	0.046	31 (70.5%)		13 (29.5%)	0.225
No	140 (57.1%)	105 (42.9%)		149 (60.8%)		96 (39.2%)	
No. of Pharmacist per Pharmacy							
<2	131 (53.9%)	112 (46.1%)	0.550	156 (64.2%)		87 (35.8%)	0.123
>2	27 (58.7%)	19 (41.3%)		24 (52.2%)		22 (47.8%)	
Customer per month							
<2000	10 (11.6%)	76 (88.4%)	0.000	60 (69.8%)		26 (30.2%)	0.088
>2000	148 (72.9%)	55 (27.1%)		120 (59.1%)		83 (40.9%)	
Customers purchasing oral anti-diabetic medications per month							
<50	8 (25.0%)	24 (75.0%)	0.000	21 (65.6%)		11 (43.4%)	0.874
51-100	8 (20.5%)	31 (79.5%)		25 (64.1%)		14 (35.9%)	
>100	142 (65.1%)	76 (34.9%)		134 (61.5%)		84 (38.5%)	
Customer Purchasing Insulin per month							
<10	8 (33.3%)	16 (66.7%)	0.000	15 (62.5%)		9 (37.5%)	0.038
Oct-50	32 (37.2%)	54 (62.8%)		63 (73.3%)		23 (26.7%)	
>50	118 (65.9%)	61 (34.1%)		102 (57.0%)		77 (43.0%)	

To find characteristics of community pharmacist and community pharmacy linked with current practice, logistic regression models were used. Community pharmacists who thought providing a service was part of their job were more inclined to do so. Community pharmacies with more than 50 diabetes patients purchasing insulin and diabetes medicine in a month (for education on diet) and engagement in the training of diabetes were also recognized as facilitators (for compliance monitoring services). The lack of pharmacist availability was highlighted as a barrier to efficiently providing diabetes and compliance monitoring services. Table 4 summarizes the odds ratios of the significant characteristics.

**Table 4:** Odds ratios of significant characteristics of pharmacist and community pharmacy with the present practices

Variables	B	OR	95% CI for OR		P-value 0.05
			Lower	Upper	
Services for Types 2 Diabetes					
Opening Hours per day					
15-20 hours per day	2.696	0.76	0.019	0.3	0.000
Age					
<25	2.967	0.051	0.007	0.362	0.003
Pharmacy Setting					
Stand-alone pharmacies	1.869	6.485	1.291	32.568	0.023
Customers purchasing oral anti-diabetic medications per month					
<50 customers per months	2.39	10.917	1.123	106.097	0.039
Customer Purchasing Insulin per month					
<10 customer per months	4.287	72.768	1.897	2792.03	0.021
Perceived Role of Community Pharmacist					
Customer Purchasing Insulin per month					
10-15 customers per month	-1.167	0.311	0.111	0.874	0.027

## Discussion

For the management of diabetic patients, proper pharmaceutical treatment is crucial to achieving the desired outcome. Community pharmacists help in controlling blood sugar levels and enhance the quality of life by providing pharmaceutical care and prescription management services [10].

This study highlights the community pharmacist’s existing practices and their role in type 2 diabetes care. As the survey requested input about future services, respondents are likely more enthused about them than they are about their implementation.

Although dispensing was firmly established in Rawalpindi and Islamabad community pharmacies, services besides dispensing were only given to a limited level. The most regular practice here was providing basic medication information on how to use them. Also, at community pharmacies in Pakistan, prescription handling and patient management are rarely seen [11].

Most of the research conducted in developed countries reported pharmacists were consistently counseling patients about their prescription drugs, including usage and adverse effects [12]. However, pharmacists have been shown to play a significant role in delivering lifestyle modification and smoking cessation education [13], assisting patients with self-monitoring blood glucose (SMBG) [12], and supervising medication adherence [3]. Less common practices have been documented, such as tracking treatment progress and engaging in treatment strategies. Despite their limited ability to provide services other than dispensing, the most of community pharmacists of Rawalpindi and Islamabad believed that their services should be expanded. Studies conducted in developed countries reflected similar results for, the preferences of pharmacists in education and monitoring services, like training about medicines, healthy lifestyles, and SMBG; monitoring of medication compliance, carrying out a test for the monitoring of blood glucose, and providing feedback on glycemic control [14]. Pharmacists can fill the vacuum left by most Pakistani physicians who do not commit enough time to counsel and educate their patients properly.

Community pharmacists are underutilized in contrast to the situation in developed countries since they are not seen as healthcare professionals and as a result, their contribution to the healthcare system is not acknowledged [15]

When a pharmacist's responsibility included services, the regression models demonstrated that this perspective acted as a catalyst for the provision of a wide range of services for patients. Indonesian research found that pharmacists who worked in pharmacies that provided diabetes care had a much greater agreement with the service than those who worked in pharmacies that did not [16].

The availability of pharmacists for various patients' education and monitoring activities was associated with their degree of availability. It is required by law that a pharmacist must be there throughout the operational hours of a community pharmacy [17]. More than 80% of pharmacies in Rawalpindi and Islamabad, on the other hand, may have had no pharmacists throughout their business hours.

Administrative barriers, insufficient implementation of rules and regulations, an insufficient number of pharmacy personnel (0.06 pharmacists for 10,000 people - 6/1,000,000), a pharmacist's academic expertise in terms of clinical services revolving around a patient, a deficiency of collaboration with other healthcare personnel, and a lack of knowledge about the existence and role of pharmacists are all obstacles to the expansion of pharmacy services in community settings [13].

Weak monitoring mechanisms and law enforcement may contribute to this, making implementation dependent on the pharmacist's dedication. Because proprietors (non-pharmacist managers' owners) own the majority of pharmacies, their dedication is likely to affect this practice. According to this study, pharmacies managed by pharmacist managers had pharmacists present throughout business hours than pharmacies owned by non-pharmacist owners. Low pharmacist availability might indicate that owners are more focused on business, selling drugs in less expensive methods without the use of pharmacists. Many pharmacists would be employed only for legal reasons, which would result in reduced salaries. Pharmacists may take on other jobs due to low pay and shorter hours, causing them to be unavailable at pharmacies.

Despite continuous government initiatives, and literature and policy that demand pharmacy services to be implemented, deficiency of acknowledgment of the entire breadth of pharmacy services has limited pharmacists' ability to establish themselves as skilled and acknowledged healthcare providers in Pakistan [10]. Along with a few exceptions, advanced patient-centered pharmacy services are not being provided in Pakistani hospitals and private-sector pharmacies [18].

The general public underutilizes community pharmacies, which may make it difficult for pharmacy owners to provide services like pharmacist availability. Each month, the majority of local pharmacies claimed to serve over 2000 people. Pharmacist involvement in diabetes education was found to be a useful tool for delivering teaching and monitoring duties. Pharmacists who have received diabetes training execute more diabetes-related activities than pharmacists who have not received diabetes training, according to studies conducted in Australia and Canada [12,19]. As a result, Pakistani pharmacy councils may consider offering formal diabetes training to improve the skills of pharmacy graduates. Pharmacies with the designated counseling area, those in clinics, and those with more diabetes patients were all more likely to engage in various teaching and monitoring activities. Despite this, only 15.2% of pharmacies have a counseling space or room. Pharmacies within clinics, according to the findings, may create chances for pharmacists to develop professional relations with physicians, hence increasing the establishment of additional diabetes services. The vicinity of practice places was one of the facilitators for forming solid cooperation. Furthermore, increased customers may be associated with higher revenue and, as a result, the drugstore's ability to offer additional services along with the availability of pharmacists, employment of adequate staff, and training of diabetes-related services. More turnover is one of the factors in administering diabetes therapy, according to an Australian study [1].

Strengths 

The research would provide information to enable the government and pharmacy authorities to construct community pharmacy-centered diabetes care in a developing country like Pakistan.

Limitations 

The limitations include the availability of community pharmacists and a lack of published data on type 2 diabetes service coverage, use, or quality in Rawalpindi and Islamabad

## Conclusions

In Rawalpindi and Islamabad, most community pharmacies only provide a basic dispensing service for diabetes patients. Most of the community pharmacists agreed to extend their duties. The expansion of pharmacist professional responsibilities would help control the rising diabetes burden. The facilitators and hurdles identified would serve as a foundation for the introduction of diabetic care in community pharmacies.

## Appendices

Evaluation of Community Pharmacists for Type 2 Diabetes Services in Rawalpindi and  Islamabad

Questionnaire

Pharmacist Characteristics

Gender

• Male

• Female

Age _________

Year of Registration _________

Position

• Pharmacist Manager as well as owner

• Pharmacist Manager

• Pharmacist as well as QP

• Staff Pharmacist

Years of Experience as Community Pharmacist

• ≤ 5 Years

• 6-10 Years

• > 10 Years

Diabetes Training/continuing education in last year

• None

• 1-5 h

• 6-10 h

• >10 h

Pharmacy Characteristics

Setting 

• Chain Store

• Stand Alone

• Pharmacy within Shopping Mall Complex

• Pharmacy within Clinics

Ownership

• Pharmacist Manager as Owner

• Single or Group Proprietor

• Partnership Proprietor-Pharmacist Manager

Opening Days per week _________

Opening Hours per Week ________

No. of Pharmacist per Pharmacy _________

Customer per month

•≤1000•1001-2000•>2000

 Customers purchasing oral anti-diabetic medications per month 

 • ≤50 

 • 51-100 

 • >100 

 Customer Purchasing Insulin per month 

 • ≤10 

 • 10-50 

 • >50 

 Current Pharmacy-based services for Type-2 Diabetes 

 Dispensing 

 Prepare Medication 

 • Never• Very  rarely• Rarely• Occasionally• Very  Frequently• Always

 Provide Label on Direction for use

 • Never• Very  rarely• Rarely• Occasionally• Very  Frequently• Always

 Services Beyond Dispensing

 Initial Assessment

 Patient history

 • Never• Very  rarely• Rarely• Occasionally• Very  Frequently• Always

 Baseline physical examination (e. g. measure weight/height, blood pressure)

 • Never• Very  rarely• Rarely• Occasionally• Very  Frequently• Always

 Baseline test (e. g. check blood glucose)

 • Never• Very  rarely• Rarely• Occasionally• Very  Frequently• Always

 Treatment plan

 • Never• Very  rarely• Rarely• Occasionally• Very  Frequently• Always

 Patient Education

 Disease process

 • Never• Very  rarely• Rarely• Occasionally• Very  Frequently• Always

 Treatment targets

 • Never• Very  rarely• Rarely• Occasionally• Very  Frequently• Always

 Anti-Diabetic Medications:

 Directions for use

 • Never• Very  rarely• Rarely• Occasionally• Very  Frequently• Always

Use of insulin devices

• Never• Very  rarely• Rarely• Occasionally• Very  Frequently• Always

Storage requirements

• Never• Very  rarely• Rarely• Occasionally• Very  Frequently• Always

Special precautions to follow

• Never• Very  rarely• Rarely• Occasionally• Very  Frequently• Always

Common/important adverse effects

• Never• Very  rarely• Rarely• Occasionally• Very  Frequently• Always

Exercise

• Never• Very  rarely• Rarely• Occasionally• Very  Frequently• Always

Diet Plan

• Never• Very  rarely• Rarely• Occasionally• Very  Frequently• Always

Self-monitoring of blood glucose (SMBG)

• Never• Very  rarely• Rarely• Occasionally• Very  Frequently• Always

Prevention/treatment of acute complications

• Never• Very  rarely• Rarely• Occasionally• Very  Frequently• Always

Prevention/treatment of chronic complications

• Never• Very  rarely• Rarely• Occasionally• Very  Frequently• Always

Needs for regular medical monitoring

• Never• Very  rarely• Rarely• Occasionally• Very  Frequently• Always

Foot self-care

• Never• Very  rarely• Rarely• Occasionally• Very  Frequently• Always

Smoking cessation

• Never• Very  rarely• Rarely• Occasionally• Very  Frequently• Always

Monitoring

Monitor compliance with:

Antidiabetic medications

• Never• Very  rarely• Rarely• Occasionally• Very  Frequently• Always

Exercise plan

• Never• Very  rarely• Rarely• Occasionally• Very  Frequently• Always

Diet plan

• Never• Very  rarely• Rarely• Occasionally• Very  Frequently• Always

Plan for prevention/treatment of chronic complications

• Never• Very  rarely• Rarely• Occasionally• Very  Frequently• Always

Scheduled medical monitoring

• Never• Very  rarely• Rarely• Occasionally• Very  Frequently• Always

Monitor Treatment Outcomes:

Check records on SMBG

• Never• Very  rarely• Rarely• Occasionally• Very  Frequently• Always

Carry out blood glucose tests

• Never• Very  rarely• Rarely• Occasionally• Very  Frequently• Always

Measure BMI

• Never• Very  rarely• Rarely• Occasionally• Very  Frequently• Always

Measure blood pressure

• Never• Very  rarely• Rarely• Occasionally• Very  Frequently• Always

Check results on laboratory tests

• Never• Very  rarely• Rarely• Occasionally• Very  Frequently• Always

Monitor for adverse effects

• Never• Very  rarely• Rarely• Occasionally• Very  Frequently• Always

Review

• Never• Very  rarely• Rarely• Occasionally• Very Frequently• Always

Perceived role of Pharmacist for Type-2 Diabetes

Dispensing

Prepare Medication

• Definitely No• No• Probably No• Probably Yes• Yes• Definitely  Yes 

Provide Label on Direction for use

• Definitely No• No• Probably No• Probably Yes• Yes• Definitely  Yes 

Services Beyond Dispensing

Initial Assessment

Patient history

 • Definitely No• No• Probably No• Probably Yes• Yes• Definitely  Yes

Baseline physical examination (e. g. measure weight/height, blood pressure)

 • Definitely No• No• Probably No• Probably Yes• Yes• Definitely  Yes

Baseline test (e. g. check blood glucose)

 • Definitely No• No• Probably No• Probably Yes• Yes• Definitely  Yes

Treatment plan

 • Definitely No• No• Probably No• Probably  Yes• Yes• Definitely  Yes

 Patient Education

 Disease process

 • Definitely No• No• Probably No• Probably Yes• Yes• Definitely  Yes

Treatment targets

 • Definitely No• No• Probably No• Probably Yes• Yes• Definitely Yes

 Anti-Diabetic Medications:

 Directions for use

 • Definitely  No• No• Probably  No• Probably  Yes• Yes• Definitely  Yes

Use of insulin devices

 • Definitely  No• No• Probably  No• Probably  Yes• Yes• Definitely  Yes

Storage requirements

 • Definitely  No• No• Probably  No• Probably  Yes• Yes• Definitely  Yes

Special precautions to follow

 • Definitely  No• No• Probably  No• Probably  Yes• Yes• Definitely  Yes

Common/important adverse effects

 • Definitely  No• No• Probably  No• Probably  Yes• Yes• Definitely  Yes

Exercise

 • Definitely  No• No• Probably  No• Probably  Yes• Yes• Definitely  Yes

Diet Plan

 • Definitely  No• No• Probably  No• Probably  Yes• Yes• Definitely  Yes

Self-monitoring of blood glucose (SMBG)

 • Definitely  No• No• Probably  No• Probably  Yes• Yes• Definitely  Yes

Prevention/treatment of acute complications

 • Definitely  No• No• Probably  No• Probably  Yes• Yes• Definitely  Yes

Prevention/treatment of chronic complications

 • Definitely  No• No• Probably  No• Probably  Yes• Yes• Definitely  Yes

Needs for regular medical monitoring

 • Definitely  No• No• Probably  No• Probably  Yes• Yes• Definitely  Yes

Foot self-care

 • Definitely  No• No• Probably  No• Probably  Yes• Yes• Definitely  Yes

Smoking cessation

 • Definitely  No• No• Probably  No• Probably  Yes• Yes• Definitely  Yes

Monitoring

Monitor compliance with:

Antidiabetic medications

• Definitely  No• No• Probably  No• Probably  Yes• Yes• Definitely  Yes

Exercise plan

• Definitely  No• No• Probably  No• Probably  Yes• Yes• Definitely  Yes

Diet plan

• Definitely  No• No• Probably  No• Probably  Yes• Yes• Definitely  Yes

Plan for prevention/treatment of chronic complications

• Definitely  No• No• Probably  No• Probably  Yes• Yes• Definitely  Yes

Scheduled medical monitoring

• Definitely  No• No• Probably  No• Probably  Yes• Yes• Definitely  Yes

Monitor Treatment Outcomes:

Check records on SMBG

• Definitely  No• No• Probably  No• Probably  Yes• Yes• Definitely  Yes

Carry out blood glucose tests

• Definitely  No• No• Probably  No• Probably  Yes• Yes• Definitely  Yes

Measure BMI

• Definitely  No• No• Probably  No• Probably  Yes• Yes• Definitely  Yes

Measure blood pressure

• Definitely  No• No• Probably No• Probably  Yes• Yes• Definitely  Yes

Check results on laboratory tests

• Definitely  No• No• Probably  No• Probably  Yes• Yes• Definitely  Yes

Monitor for adverse effects

• Definitely  No• No• Probably  No• Probably  Yes• Yes• Definitely  Yes

Review

• Definitely No• No• Probably No• Probably  Yes• Yes• Definitely  Yes
